# *Bartonella quintana* in Body Lice from Scalp Hair of Homeless Persons, France

**DOI:** 10.3201/eid2005.131242

**Published:** 2014-05

**Authors:** Rezak Drali, Abdoul Karim Sangaré, Amina Boutellis, Emmanouil Angelakis, Aurélie Veracx, Cristina Socolovschi, Philippe Brouqui, Didier Raoult

**Affiliations:** Aix Marseille Université, Marseille, France; and Institut Hospitalo-Universitaire Méditerranée Infection, 13005, Marseille

**Keywords:** *Bartonella quintana*, *Pediculus humanus*, louse, head lice, scalp, hair, clothing lice, nits, louse lineage, parasites, arthropods, vector-borne, dual infestation, homeless, bacteria, trench fever, bacillary angiomatosis, endocarditis, chronic bacteremia, chronic lymphadenopathy

**To the Editor:** *Bartonella quintana* is a body louse–borne human pathogen that can cause trench fever, bacillary angiomatosis, endocarditis, chronic bacteremia, and chronic lymphadenopathy (*1*). Recently, *B. quintana* DNA was detected in lice collected from the heads of poor and homeless persons from the United States, Nepal, Senegal, Ethiopia, and the Democratic Republic of the Congo and in nits in France (*2*,*3*). The head louse, *Pediculus humanus capitis*, and the body louse, *Pediculus humanus humanus,* are obligatory ectoparasites that feed exclusively on human blood (*4*). Outside of their habitats, the 2 ecotypes are morphologically indistinguishable (*1*). Sequence variation in the PHUM540560 gene discriminates between head and body lice by determining the genotype of the lice (*5*). While surveying for trench fever among homeless persons in shelters in Marseille, France during October 2012–March 2013, we investigated the presence of *B. quintana* DNA in nits, larvae, and adult lice collected from mono-infested and dually infested persons and determined the genotypes of the specimens.

The persons included in this study received long-lasting insecticide-treated underwear; lice were collected by removing them from clothing, including underwear, pants, and shirts. Because body lice reside in the clothing of infested persons except when feeding, they are sometimes called clothing lice.

A total of 989 specimens were tested, including 149 (83 from clothing and 66 from hair) first–instar larvae hatched in the laboratory from eggs collected from 7 dually infested persons, and 840 adult body lice collected from the clothing of 80 mono-infested patients. We included DNA isolated from 3 nits collected from the hair of a mono-infested person who had previously been confirmed as positive for *B. quintana* (*6*) (Table).

**Table:** Distribution of *Bartonella quintana* DNA in nits, larvae, and adult body lice collected from hair and clothing of homeless persons in shelters, Marseille, France, October 2012– March 2013*

Location	No. persons		No. (%) lice positive for *B. quintana* DNA	Reference
	Dually infested, n = 7	Monoinfested, n = 80		
Hair				
Nits	0	3	3 (100)	(*6*)
Hatched larvae	66	0	7 (10.60)	This study
Clothing				
Hatched larvae	83	0	5 (6.00)	This study
Adults	0	840	174 (20.70)	This study

*All lice were identified as body lice. Study participants were provided with long-lasting insecticide-treated underwear, and killed body lice were collected from the clothing of infested persons.

Total DNA was extracted by using an EZ1 automated extractor (QIAGEN, Courtaboeuf, France) and subjected twice to real-time PCR specific for *B. quintana*. The first PCR targeted the 16S-23S intergenic spacer region. Positive samples were confirmed by using a second real-time PCR targeting the *yopP* gene (*6*). Samples that tested positive for *B. quintana* DNA were analyzed by multiplex real-time PCR that targeted the PHUM540560 gene (*5*). We used head and body lice that had known genotypes positive controls. Negative controls were included in each assay.

Of the hatched larvae, 5 (6%) of the 83 recovered from clothing and 7 (11%) of 66 from the hair (Table) of 4 of the 7 dually infested persons were positive for *B. quintana* DNA (Technical Appendix). Of the 840 adult body lice, 174 (21%) collected from 42 (53%) of 80 of the mono-infested persons contained *B. quintana* DNA (Table, Technical Appendix). The multiplex real-time PCR that targeted the PHUM540560 gene clearly identified all nits, larvae, and adult lice as belonging to the body lice lineage. Negative controls remained negative in all PCR-based experiments.

For 2 decades, *B. quintana* DNA has been regularly detected in lice collected from the heads of persons living in poverty, but it had not been detected in head lice that infest schoolchildren (*7*,*8*). All of the lice collected during this study that tested positive for *B. quintana* from homeless persons were body lice, including some that were recovered from hair. This observation supports our assertion that body lice are not confined to the body. The 3 eggs that were removed from the hair of a mono-infested homeless person whose samples tested positive for *B. quintana* were also body lice. During the clinical examination, no adult head lice or adult body lice were found on that person, confirming that the patient had been heavily infested with body lice in the past, not head lice. The nits were most likely laid by body lice that migrated toward the patient’s head. When a member of this research team (DR) collected the eggs from the hair shaft, they were found ≈3–3.5 cm from the hair follicle. Because hair grows ≈1.25 cm per month, the louse infestation occurred ≈3 months before egg collection (*6*).

Homeless persons that we have monitored for many years are often heavily infested by body lice but are also occasionally infested with head lice. Before genetic tools that differentiate the head and body louse lineages were available (*5*), it was speculated that body lice may have originated from head lice (*9*). From our study, it is clear that under conditions of massive infestation, body lice can migrate and colonize hair; the opposite may also be true. However, there is no evidence that body lice are capable of causing an outbreak of lice living on the head, as happens among schoolchildren that have been found to be infested only by head lice. This suggests that body lice cannot thrive in the environment of head lice, which infest millions of children worldwide (*10*), further suggesting that outbreaks of trench fever are most likely not linked to head lice in industrialized countries. In conclusion, by analyzing lice harvested from the heads and clothing of homeless persons, we have shown that the 2 ecotypes belong to the same body lice population.

Technical Appendix Detailed distribution of *B. quintana* DNA among lice from mono-infested and dually infested homeless persons, France
