# Supplementary material for: Bartonella quintana in Body Lice from Scalp Hair of Homeless Persons, France
Source: Emerg Infect Dis. 2014 May;20(5):907–8. doi: 10.3201/eid2005.131242 (PMC4012801; doi:10.3201/eid2005.131242)
Supplement: Technical Appendix — Detailed distribution of B. quintana DNA among lice from mono-infested and dually infested homeless persons, France [file 13-1242-Techapp-s1.pdf]

Article DOI: http://dx.doi.org/10.3201/eid2005.131242

Bartonella quintana in Body Lice from Scalp Hair of Homeless Persons, France

Technical Appendix

Detailed distribution of *B. quintana* DNA among lice from mono-infested and dually infested homeless persons, France

Technical Appendix Table 1: Distribution of *B. quintana* DNA among lice from dually infested homeless persons, France, October 2012–March 2013

	Lice collected from body		Lice collected from head	
ID code homeless persons	Lice tested	*B. quintana* no. (%)	Lice tested	*B. quintana* no. (%)
32	5	0	4	0
33	5	2 (40.00)	2	1 (50.00)
40	5	1 (20.00)	4	0
89	29	0	6	0
B	17	0	30	5 (16.60)
D	11	2 (18.20)	10	1 (10.00)
Nov	11	0	10	0
Total	83	5 (6.00)	66	7 (10.60)

Technical Appendix Table 2: Detail of distribution of *B. quintana* DNA among lice from mono-infested homeless persons, France, October 2012–March 2013

ID no. homeless persons	Body lice	*B. quintana*
1	3	0
2	3	1
3	3	3
4	3	0
5	3	0
6	3	0
7	3	3
8	3	3
1001	69	13
1002	17	7
1003	18	6
1005	3	0
1022	10	0
1023	3	3
1029	15	9
1034	3	1
1037	8	2
1038	101	3
1040	5	1
1051	2	0
1052	74	15
1053	19	2
1054	3	1
1059	19	0
1060	3	3
1065	4	2
1066	2	1
1070	7	3
1087	23	4
1101	2	2
1105	1	0

ID no. homeless persons	Body lice	*B. quintana*
1151	10	5
1152	17	2
1153	1	0
1155	6	2
1158	4	0
1159	5	4
1160	3	0
1161	4	0
1163	1	1
1200	9	4
1201	24	0
1202	1	0
1203	1	0
1204	2	0
1205	1	0
1206	5	0
1208	21	2
1209	2	2
1210	23	13
1211	16	10
1212	5	1
1213	4	0
1222	46	1
1223	28	5
1224	2	0
1225	2	0
1227	2	0
1228	2	0
1229	18	3
1230	2	0
1231	6	1
1232	2	0
1234	10	1
1236	4	0
1237	44	19
1238	2	0
1239	2	0
1240	11	5
1241	1	1
1242	5	4
1244	2	0
1245	3	0
1246	7	0
2002	1	0
2016	34	0
2040	1	0
2101	1	0
2103	2	0
Total (%)	840	174 (20.7)
